# Activated Carbon for Drug Delivery from Composite Biomaterials: The Effect of Grinding on Sirolimus Binding and Release

**DOI:** 10.3390/pharmaceutics14071386

**Published:** 2022-06-30

**Authors:** Zhanna K. Nazarkina, Tatyana A. Savostyanova, Boris P. Chelobanov, Irina V. Romanova, Pavel A. Simonov, Ren I. Kvon, Andrey A. Karpenko, Pavel P. Laktionov

**Affiliations:** 1 Institute of Chemical Biology and Fundamental Medicine, Siberian Branch, Russian Academy of Sciences, 630090 Novosibirsk, Russia; t.savostyanova@g.nsu.ru (T.A.S.); boris.p.chelobanov@gmail.com (B.P.C.); irin-romanova@yandex.ru (I.V.R.); lakt@niboch.nsc.ru (P.P.L.); 2 Department of Natural Sciences, Novosibirsk State University, 630090 Novosibirsk, Russia; spa@catalysis.ru; 3 Boreskov Institute of Catalysis, Siberian Branch, Russian Academy of Sciences, 630090 Novosibirsk, Russia; kvon@catalysis.ru; 4 Meshalkin National Medical Research Center, Ministry of Health of the Russian Federation, 630055 Novosibirsk, Russia; andreikarpenko@rambler.ru

**Keywords:** activated carbon, drug delivery, controlled release, electrospinning, sirolimus

## Abstract

Activated carbon (AC) could be potentially useful as a drug carrier in fiber polymer scaffolds destined for prolonged drug delivery. To be introduced, AC must be ground into smaller-sized particles to be introduced in scaffolds, as most biocompatible scaffolds consist of fibers with a diameter of less than 1 µm. In this study, the adsorption of sirolimus (SRL) from phosphate-buffered saline (PBS) solution and blood plasma (BP) onto AC of AX-21 type, as well as the release of SRL from AC depending on its fragmentation, were studied. Two-stage grinding of the AC, first with a ball mill, and then with a bead mill, was performed. Grinding with a bead mill was performed either in water or in polyvinylpyrrolidone to prevent aggregation of AC particles. Dynamic light scattering and scanning electron microscopy (SEM) demonstrated that the size of the particles obtained after grinding with a ball mill was 100–10,000 nm, and after grinding with a bead mill, 100–300 nm. Adsorption in PBS was significantly higher than in BP for all fractions, and depended on SRL concentration. The fraction obtained after grinding with a ball mill showed maximal SRL adsorption, both in PBS and BP, and slow SRL release, in comparison with other fractions. The 100–300 nm AC fractions were able to adsorb and completely release SRL into BP, in contrast to other fractions, which strongly bound a significant amount of SRL. The data obtained are to be used for controlled SRL delivery, and thus in the modification of drug delivery in biological media.

## 1. Introduction

Activated carbon (AC) is a strong and efficient binder of drugs and toxic compounds. AC is used for drinking water purification, and as an antidote to eliminate poisoning in clinical practice [1,2,3]. It is widely used in the cosmetic industry and in medicine, not only for oral hygiene [4], skin application [5], and as an oral drug [6], but also for hemoperfusion using an activated-carbon-coated column [7]. AC is non-toxic, and possesses good bio- and hemocompatibility. This offers the possibility for AC to be used not only for the adsorption of drugs, but also for the release of pre-adsorbed drugs [8,9], which allows accurate tuning of their concentration and exposure time. AC could be oxidized and modified to improve the adsorption capacity and change the affinity [10]. Jandosov and colleagues developed a microgranulated binary biomedical preparation consisting of a pectin core-shell and nanoporous AC as the shell, for oral use [11]. This composite material demonstrated substantial efficiency for removing lead (II) nitrate and sodium diclofenac. In addition to products based on granular and powder AC, activated carbon cloth (ACC) is also used in the medical field. ACC is obtained via carbonization and the activation processes of precursor fibers. ACC is used for wound care, including antimicrobial and anti-odor dressings, and negative wound therapy filters [12]. Olivier and colleagues explored the potential of combining activated carbon fiber cloth and calcium-deficient hydroxyapatite (CDA) for use as a dual drug delivery system for bone regeneration [13]. A significant acceleration of bone reconstruction in rats was observed in the presence of the ACC/CDA patch. Aspirin-loaded ACC/CDA material has been shown to be able to release the drug in situ to improve bone healing.

Adsorption properties of AC could be potentially used for targeting drug delivery. For example, previously, electrospun (ES) sirolimus (SRL) and paclitaxel-enriched matrices have been proposed for drug-eluting stent coating [14,15]. It was demonstrated that the artery wall effectively retains the drug released from the coating, and can reduce the required dose of the drug [16]. However, these stent coatings also release drugs into the blood, and the prevention of their release into circulation could be achieved by drug adsorption by an AC-enriched layer introduced into such coatings. A layer of porous material that sorbs the drug can be used for vectored controlled drug release, and the prevention of drug loss into the blood. The materials applied should be stable, safe for the body, have a good affinity for the drug, and release the drug in a controlled manner.

Prolonged drug delivery can also be executed using drug-loaded AC, either used alone or packed in polymers, such as films, foams, and fibers. Packaging in fibers requires the AC to be disintegrated into small particles. For example, ES fibers are in the range of 0.5 to 2 microns, and thus, at least 0.1- to 0.3-micron AC particles are of interest. It should be noted that AC-enriched ES materials can also be used for drug delivery, not only in clinics, but also for water/solvent purification, air cleaning, etc. Packing of AC into fibers produced by electrospinning makes it possible to eliminate any contact of the AC with biomolecules, whose size exceeds the pore size of the fibers, and thus, will make it possible to obtain an adsorbent that is fundamentally different from AC simply introduced into biological media. Moreover, the introduction of AC in such way can enhance the physical properties of such fibers, and eliminate any entrance of AC into the organism if stable polymers are used for electrospinning, thus eliminating any concerns related to nanoparticle–cell interactions. At the same time, the grinding of AC can change the number and nature of functional groups, and interfere with the structure of the carbon (e.g., change in the number of pores of a certain size), which, together, could interfere with AC binding capacity. These mechanical effects on solids lead to the formation of a new surface that interacts with environmental components. This interaction causes chemical modification of the new surface. These processes are valid for carbon materials [17].

Sirolimus (SRL), also known as rapamycin, was isolated from *Streptomyces hygroscopicus*, collected from Easter Island [18] as an antifungal agent. Subsequent studies revealed antitumor, immunosuppressive, antiproliferative, antiangiogenic, antifungal, anti-restenosis, and anti-inflammatory properties of SRL [19]. The molecular mechanisms underlying the antifungal, antiproliferative, and immunosuppressive activities are the same [18]. SRL forms a complex with an abundant immunophilin, FKBP12. This complex interacts with and inhibits the activity of the cell-cycle–specific kinase, mTOR, which is involved in a number of cellular processes, such as cell growth and proliferation, immunity, angiogenesis, fibrogenesis, etc. The inactivation of mTOR leads to cycle arrest at the G1 to S phase. A number of studies have demonstrated that SRL can be used for treating various diseases: cancer, neurodegenerative and metabolic disorders, vascular restenosis, and organ transplantation [19]. SRL is a hydrophobic molecule (https://pubchem.ncbi.nlm.nih.gov/compound/5284616 (accessed on 17 June 2022)) with a molecular weight of 914  g/mol, and has poor aqueous solubility (2.6 µg/mL) [20], which has thus far hampered its clinical application. SRL delivery is still acknowledged and confirmed by recent studies of various nanostructured carriers, such as liposomes, micelles, polymeric nanoparticles, nanocrystals, magnetic nanoparticles, albumin nanoparticles, solid dispersion nanoparticles, and niosomes for SRL delivery [19]. Release of SRL from electrospinning-produced matrices in different medias was studied, and more efficient release in blood plasma than in phosphate buffer saline was observed [15].

This current study investigated the application of AC as a drug adsorbent for vectored controlled drug delivery. The adsorption of a large number of drugs on different AC variants has been studied previously; however, to date, no data on the adsorption of SRL onto AC have been published. The properties of different fractions of activated carbon after grinding with a ball mill and subsequently with a bead mill were studied. The influence of size of the carbon particles on SRL loading onto AC, and its release into different solutions, was investigated.

## 2. Materials and Methods

### 2.1. Production of ^3^H-SRL

Tritium-labeled sirolimus (^3^H-SRL) was synthesized by thermoactivated tritium exchange, as described previously [21], using SRL from Fujian Kerui Pharmaceutical Co., Ltd., (Fujian 150301, China). ^3^H-SRL was purified after labeling, and the radioactivity of the samples was evaluated as reported in [15].

### 2.2. Preparation of AC

For the purpose of the present work, active carbon AX-21 (Anderson Development Company, Adrian, MI, USA) was chosen. It is presumably microporous, with surface area determined by the Brunauer–Emmett–Teller method (BET) and pore volume being 2330 m^2^/g and 1.7 cm^3^/g, respectively [22]. Different powder fractions of AX-21 were tested. To obtain fraction C1 with particle size of about 0.5 microns, the original AX-21 (fraction C0) was ground with a ball mill (PULVERISETTE 6 Fritsch GmbH, Idar-Oberstein, Germany) using 50 balls of 1 cm in diameter, agate glass lining, at 400 rpm, 8 trials for 15 min each, with cooling in between milling. The loading of the mill with carbon with an initial particle size of less than 100 microns was 6–7 g. For further grinding, fraction C1 was suspended in water or aqueous polyvinylpyrrolidone (PVP, 0.68% by weight), and then ground with a bead mill to obtain fractions CW2 and CP2, respectively (particle size of about 0.1 microns). The bead mill (Stirred Bead Mill, SP-10.5W, Shenzhen Sanxing Feirong Machine Co., Ltd., Shenzhen, China) was loaded with 1390 g of ceramic beads (0.25 ÷ 0.35 mm) and 11 g of C1 in 350 mL of water or PVP solution. Grinding was performed for 1.5 h at 2400 rpm. At the end of the process, the suspensions were left, without stirring, overnight, and the supernatant was collected and lyophilized. The samples of lyophilized carbon were weighed and resuspended in water to obtain suspensions with concentrations of 1 mg/mL.

### 2.3. Characterization of the Activated Carbon

#### 2.3.1. Dynamic Light Scattering

The particle size was determined using a Zetasizer Nano Z (Malvern Instruments Ltd., Malvern, UK).

#### 2.3.2. SEM Analysis

The surface microstructure of carbon particles was studied by scanning electron microscopy (SEM). The carbon particles were fixed on a sample holder using double-sided carbon tape and analyzed using an EVO 10 scanning electron microscope (Carl Zeiss AG, Oberkochen, Germany) at an accelerating voltage of 10 kV.

#### 2.3.3. X-ray Photoelectron Spectroscopy

X-ray photoelectron spectroscopy (XPS) was performed using a SPECS electron spectrometer equipped with a PHOIBOS-150 MCD-9 hemispherical analyzer and a non-monochromatic MgK source (SPECS GmbH, Berlin/Heidelberg Germany) as described previously [23].

### 2.4. SRL Adsorption and Release Kinetics

The kinetics of SRL adsorption onto AC in PBS and blood plasma was studied at different SRL concentrations. The work was approved by the Local Ethical Committee of the Center of Personalized Medicine ICBFM SB RAS (No 8, 7 July 2020). ^3^H-SRL was combined with unlabeled SRL to obtain the preparations, with radioactivity of ~3 × 10^−6^ Ci/mg. The mixtures contained 200 μg of carbon and 10 μg of SRL (low SRL concentrations) or 100 μg of carbon and 90 μg of SRL (high SRL concentrations) and PBS or blood plasma, to a final volume of 1 mL. The mixtures were incubated at room temperature with constant stirring for 30 min, 1 h, 3 h, 8 h, 24 h, and 48 h. At each time point, mixtures were centrifuged for 8 min at 12,000× *g*, after which, aliquots of 50 μL were taken and mixtures were resuspended and incubated until the next time point. The radioactivity of the aliquots was measured in duplicate, accounting for volume changes.

After 48 h of incubation, supernatant was removed, carbon was gently washed with water, and 1 mL of fresh PBS or BP was added to each tube. The aliquots were taken after 30 min, 1 h, 3 h, 8 h, 24 h, and 48 h to study SRL release kinetics.

SRL release data were fitted to kinetic models including zero-order (percentage of SRL release versus time), first-order (log of the percentage of remaining SRL versus time), Higuchi (percentage of SRL release versus square root of time), and Korsmeyer–Peppas (log of the percentage of SRL release versus log time) models [3,24]. The square of correlation coefficient (*r*^2^) was calculated to determine which model fitted the release profile.

### 2.5. Adsorption Isotherms of SRL on Different Fractions of AC

To obtain adsorption isotherms of SRL on different fractions of carbon particles, the samples were incubated with different SRL concentrations for 15 h. The mixtures contained 33 μg of carbon and 0.05, 0.5, 1, 2.5, 10, and 20 μg of ^3^H-SRL (low SRL concentration for Freundlich model) or 20, 60, 90, 150, and 220 μg of ^3^H-SRL (high SRL concentration for Langmuir model) in 0.5 mL PBS or BP. Then, mixtures were centrifuged for 8 min at 12,000× *g*, and the radioactivity of the supernatants was measured in duplicate.

We used the Freundlich and Langmuir models [25] to analyze the adsorption data. The amount of SRL adsorbed at equilibrium per unit weight of carbon, *q* (mg/g), was calculated as follows:

(1) $$q=\frac{\left(C_{0}-C\right)}{m}*V$$

where *C*_0_ and *C* are the initial and equilibrium concentrations of SRL (mg/L), respectively; *m* is the mass of carbon (g); and *V* is the volume of solution (L).

The linear form of the Langmuir equation is:

(2) $$\frac{1}{q}=\frac{1}{q_{max}}+\frac{1}{q_{max}*K_{L}*C}$$

where *q* is the amount of SRL adsorbed onto the carbon at equilibrium (mg/g); *C* is equilibrium concentrations of SRL (mg/L); *q_max_* is the maximum adsorption capacity of the carbon (mg/g); and *K_L_* is the Langmuir constant (L/g).

The linear form of the Freundlich equation is:

(3) $$\ln q=\ln K_{F}+\frac{1}{n}\ln C$$

where *q* is the amount of SRL adsorbed onto the carbon at equilibrium (mg/g); *C* is the equilibrium concentrations of SRL (mg/L); and *K_F_* and *n* are the Freundlich constants.

The parameters *q_max_*, *K_L_*, *K_F_*, and 1/*n* were calculated from the plots of 1/*q* against 1/*C* for the Langmuir isotherm, and *lnq* against *lnC* for the Freundlich isotherm. The square of correlation coefficient *r*^2^ was calculated to determine the fitting of the models.

## 3. Results and Discussion

### 3.1. Characterization of the Activated Carbon

**Figure 1:**
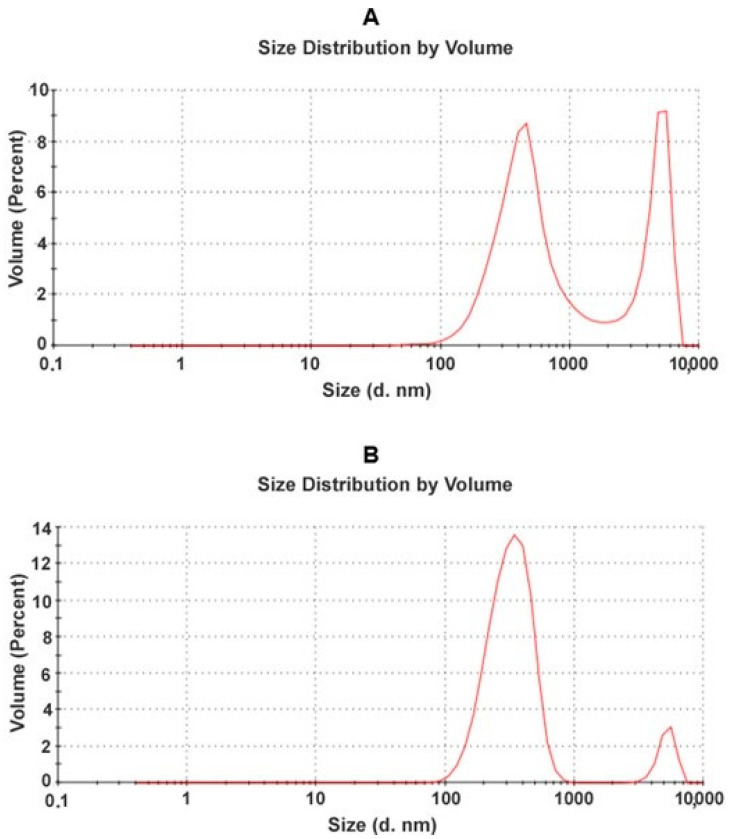
Carbon particle size distribution according to dynamic light scattering data: (**A**) after grinding AX-21 with a ball mill (fraction C1); (**B**) after 40 min of grinding C1 with a bead mill (fraction CW2).

**Figure 2:**
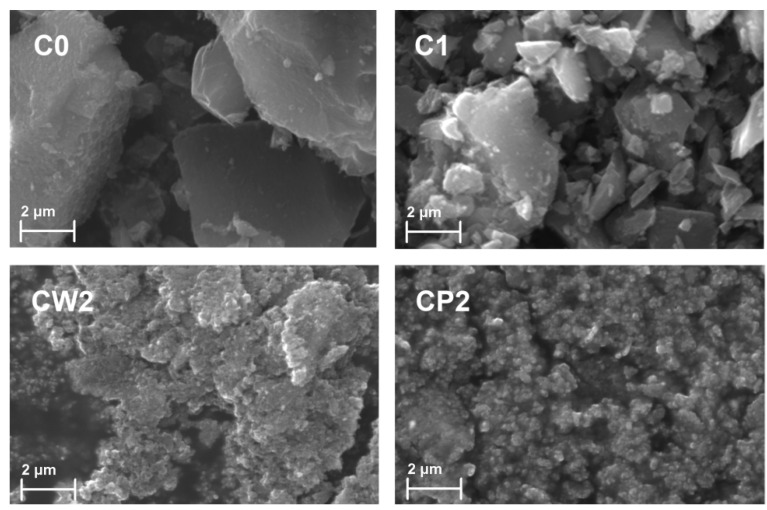
Carbon particle size according to SEM data. SEM images of AC fractions: the original AX-21 carbon (C0), the fraction after grinding with a ball mill (C1), the fraction after 40 min of grinding with a bead mill in water (CW2), and the fraction after 40 min of grinding with a bead mill in PVP (CP2).

Several types of activated carbon were compared in preliminary experiments (data not shown), and AX-21 was selected as the best and most selective binder of sirolimus from human serum. This is most likely due to it having the highest BET surface area, and many pores less than 2 nm in size, which allows multi-site van der Waals binding of the drug molecules. In order to pack AC into electrospun fibers, the carbon particle sizes must be much smaller than the fiber diameter, which is generally in the range 0.5–1 µm. To obtain AC with a particle size around 100 nm, two-stage grinding of the pristine AX-21 carbon (fraction C0) with a ball mill (to form fraction C1) and then a bead mill, was performed. Grinding was performed either in water or in polyvinylpyrrolidone (PVP) to prevent the aggregation of carbon particles. Polyvinylpyrrolidone was used as a stabilizer for the carbon suspension because of its good stabilizing properties, as well as its high biocompatibility [26]. CW2 and CP2 AC samples were obtained after bead mill grinding of the initial C1 fraction in water or in PVP. The size of the AC particles during the grinding process was determined by dynamic light scattering and SEM analysis (Figure 1 and Figure 2).

According to dynamic light scattering data, grinding with a bead mill resulted in the disappearance of particles larger than 1000 nm (Figure 1B). The main peak corresponds to particles of 100–1000 nm. Using this method, it is impossible to distinguish aggregates of small carbon particles from the large particles. We conducted SEM analysis to address this. According to SEM data, fractions CW2 and CP2 were composed of particles 100–300 nm and their aggregates (Figure 2). Grinding with a bead mill significantly decreased the size of the carbon particles; however, such particles are able to aggregate, whereas PVP prevents the aggregation of small AC particles.

**Figure 3:**
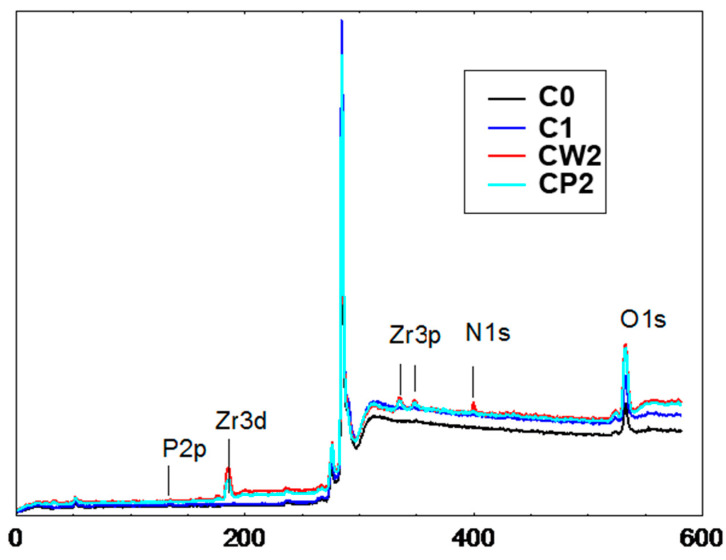
X-ray photoelectron spectroscopy of different AC fractions.

The surface chemical state of the carbon was studied using XPS (Figure 3, Table 1). The fractions C0 and C1 had similar characteristics. Grinding with the ball mill only decreased the size of the AC particles, without inducing a significant change in the concentrations of surface functional groups. Grinding with the bead mill resulted in an increase in the number of oxygen functional groups on the surface of the carbon particles. As the grinding was carried out using ceramic zirconium beads, there are peaks corresponding to zirconium in the spectra of the CW2 and CP2 AC samples. The zirconium probably entered the samples as traces of ceramics on the AC particles. This fact should be taken into account if AC particles are planned to be used in biological systems in vitro or in vivo. An increase in the concentration of the nitrogen-containing group in fraction CP2 was also apparent due to the presence of PVP.

### 3.2. Study of SRL Adsorption

#### 3.2.1. Adsorption Kinetics

**Figure 4:**
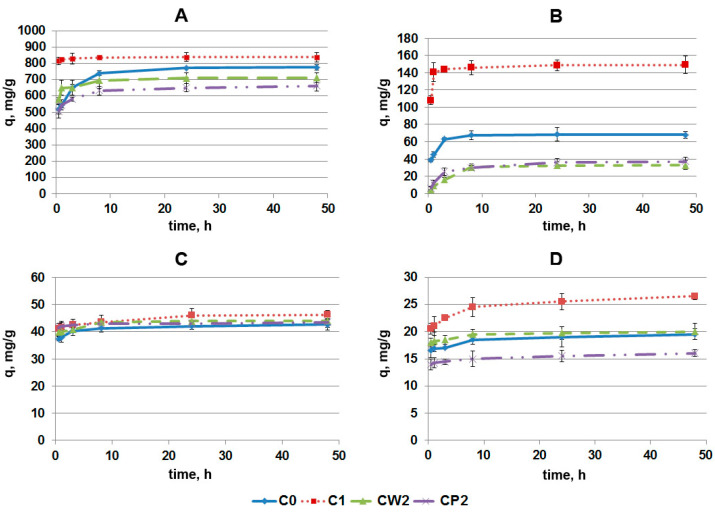
The kinetic curves of SRL adsorption onto AC. High SRL concentrations (1 mL of mixture contained 100 μg of AC and 90 μg of SRL) in PBS (**A**) and in BP (**B**). Low SRL concentrations (1 mL of mixture contained 200 μg of AC and 10 μg of SRL) in PBS (**C**) and in BP (**D**). The maximum possible SRL adsorption was 900 mg/g for (**A**,**B**) and 50 mg/g for (**C**,**D**).

Figure 4 demonstrates the influences of AC grinding and solution composition on SRL adsorption kinetics and equilibrium. At high SRL concentrations (90 µg/mL) in PBS, the bulk of the drug was adsorbed very quickly (in the first 30 min), which corresponds to the initial steep rise in the kinetic curve (Figure 4A). Then, the adsorption process slowed down, and the remaining SRL was mainly bound over the next 3 h. The nature of the kinetic curve indicates that the SRL molecules were most likely adsorbed first onto AC, near the periphery of its particles (a “fast” process), and then penetrated deeper (a “slow” process) into the pores, the average size of which was comparable to the size of the SRL molecules. This conclusion is confirmed by the fact that, for sample C1, which had a significantly smaller particle size than sample C0, the level of “fast” adsorption was higher. Nevertheless, after the incubation of SRL with AC in PBS for 20–50 h, the amount of the adsorbed drug in these samples turned out to be within the bounds of experimental error. On the contrary, for CW2 and CP2 samples obtained by grinding in a bead mill, despite having even smaller particle sizes than C1, the value of “fast” adsorption is comparable to that of the initial fraction C0, and the equilibrium SRL adsorption value was even lower. This appears to be related to the chemical state of the surface of CW2 and CP2 particles: the XPS method revealed an increased concentration of bound oxygen in these samples compared to C0 and C1 samples (Table 1).

However, at a low SRL concentration (10 µg/mL), these differences in adsorption behavior did not appear in the AC samples, and almost all of the SRL was adsorbed from the solution due to the “rapid” process on the outer surface of the AC particles (Figure 4C). This may be due to the fact that, with a small total amount of SRL input, the adsorption potential of AC is far from exhausted. It is also possible that energy heterogeneity of the adsorption centers and, at low degrees, of AC surface filling with SRL molecules, all SRL molecules only occupy the centers of strong adsorption, which may lead to the proximity of the kinetic curves of the SRL adsorption.

The strongest differences in the kinetics of SRL adsorption onto AC are observed in BP (Figure 4B,D). As in PBS, the SRL absorption was characterized by “fast” and “slow” adsorption kinetics; however, the amount of SRL adsorbed due to these processes was significantly reduced in BP compared to PBS. Thus, AC fraction C1 demonstrated the highest level of SRL adsorption: The maximal binding was 92.0% of initial SRL concentration in PBS, and 16.0% in BP (Figure 4A,B). These data demonstrate the influence of BP molecules on the rate and strength of drug binding. Thus, interaction of BP biomolecules (including bulky molecules, such as proteins, polysaccharides, proteolipids, and proteoglycans; and small molecules, such as lipids, amino acids, nucleotides, and vitamins) with AC and/or SRL impedes the adsorption of SRL on AC.

According to the intensity of absorption of SRL onto AC at the high SRL concentration (90 µg/mL) in PBS, AC fractions can be arranged in the following order: C1 > C0 > CW2~CP2, and fraction C1 and CW2/CP2 showed a 4.5-fold difference in efficiency. At low SRL concentration (90 µg/mL): C1 > CW2~C0 > CP2, with a 1.7-fold difference in efficiency for C1 and CP2.

Despite the fact that, after grinding in a bead mill, samples had smaller particle sizes and, hence, larger external surfaces, the efficiency of SRL adsorption onto CW2 and CP2 samples in PBS was lower than that of C1, and even C0; this can be attributed to a change in the chemical state of the surface of the samples after grinding in a bead mill (Table 1).

The above data show that, after 9 h of the adsorption process, equilibrium was mainly achieved for all of the fractions. For convenience, we incubated carbon particles with SRL for 15 h with different SRL concentrations, in PBS or BP, to obtain isotherms of the adsorption of SRL onto different fractions of AC.

#### 3.2.2. Adsorption Isotherms

**Figure 5:**
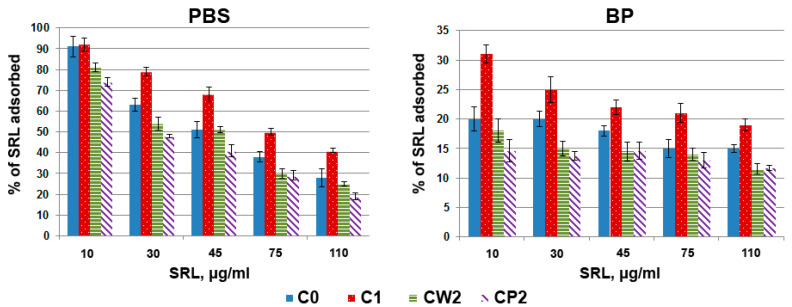
The adsorption of SRL onto different fractions of AC. Solutions of SRL with various concentrations in 0.5 mL PBS or BP were incubated with 33 μg of AC for 15 h. The percentage of adsorbed SRL relative to the initial concentration in the solution was calculated.

The data on the degree of adsorption of SRL onto different fractions of AC in PBS and BP at different initial concentrations of SRL are shown in Figure 5. All other things being equal, the maximal adsorption was observed for fraction C1 ground with a ball mill. For all fractions, adsorption in PBS was higher than in BP, which indicates the negative influence of BP components not only on the kinetics of SRL adsorption, as shown above, but also on the equilibrium of SRL adsorption. The components of BP (such as proteins and lipids) can bind SRL molecules and impede their adsorption; on the other hand, some components of BP can be adsorbed onto AC, thus blocking the adsorption sites for SRL and/or altering SRL adsorption by cooperativeness of binding. According to its adsorption capacity, AC was arranged in a row: C1 > C0 > CW2 ≥ CP2, both in PBS and BP. The increase in the adsorption capacity for sample C1 compared to C0 can be explained by an increase in the availability of the pore space of AC particles after grinding with a ball mill. According to Table 1, the chemical state of the surface of AC did not change. For fractions CW2 and CP2, we observed saturation of AC in PBS at the concentration of SRL 45 µg/mL (Figure S1). For fraction C0, the maximal adsorption in PBS was achieved at 110 µg/mL of SRL. Based on these data, the adsorption capacity for factions C0, CW2, CP2, and C1 was determined as 2, 1.3, 1.3, and >3 g of SRL per 1 g of AC, respectively. A decrease in the adsorption capacity of CW2 and CP2 samples compared to C1 and C0 was most likely caused by the fact that grinding with a bead mill leads not only to a further decrease in the size of AC particles, but also to the functionalization of their surface; the latter negatively affects the number of adsorption centers, and prevails over the effect of increasing the availability of pore space. In BP, saturation was not achieved at the maximal concentration of SRL (110 µg/mL) in all samples (Figure S1). We observed a tendency to increase adsorption, which supports the assumption that the components of BP can bind SRL molecules and impede their adsorption onto AC.

**Figure 6:**
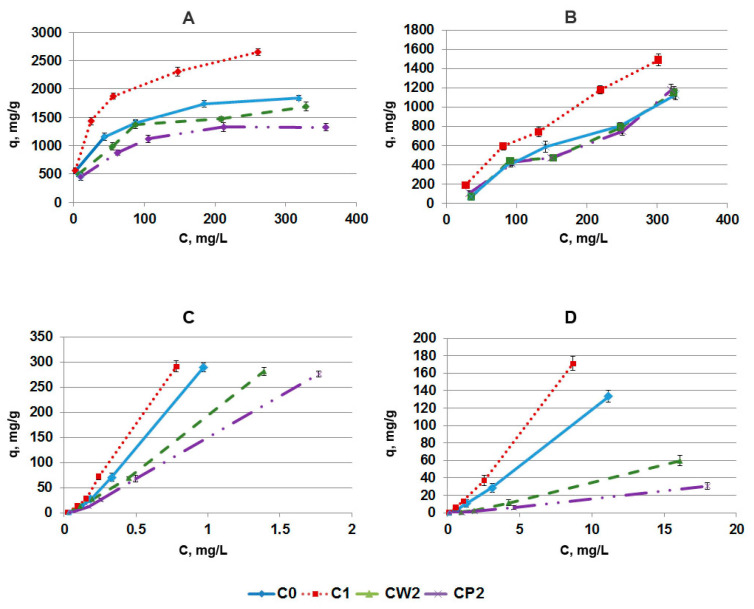
Adsorption isotherms of SRL (high concentrations) onto different fractions of AC in PBS (**A**) and BP (**B**). Adsorption isotherms of SRL (low concentrations) onto different fractions of AC in PBS (**C**) and BP (**D**).

The adsorption isotherms of SRL have complex forms (Figure 6). According to the Giles classification, adsorption isotherms refer to the S2 subgroup in PBS and the S3 subgroup in PB [27]. According to IUPAC classification, adsorption isotherms can be categorized as type V in PBS and type IV in PB [25].

We used the Freundlich and Langmuir models to analyze the isotherms of SRL adsorption onto different AC fractions (Table 2). The parameters *q_max_*, *K_L_*, *K_F_*, and 1/*n* listed in Table 2 were calculated from the plots of 1/*q* against 1/*C* for the Langmuir isotherm, and from the plots of *lnq* against *lnC* for the Freundlich isotherm. The values of 1/*n* calculated from the Freundlich isotherm were greater than 1 for all AC fractions. This means that the change in concentration of SRL affects the adsorption process significantly. This is most likely due to the energy heterogeneity of some part of the adsorption centers, which is especially pronounced at a low equilibrium concentration of SRL in solution, i.e., at low degrees of filling the surface of AC with SRL molecules. At high equilibrium concentrations of SRL, more energetically homogeneous centers are filled; therefore, on average, the adsorption equilibrium begins to correspond to the Langmuir isotherm.

The experimental values of maximal adsorption capacity, *q_max(exp_*_)_, of all AC fractions for SRL in PBS were close to those estimated theoretically *q_max(calc)_* (Table 2). The *K_L_* values in PBS remained close for fractions C0, CW2, and CP2 (for fraction C1 ground in a ball mill, the *K_L_* value was 1.6 times higher), despite the differences in the value of q_max_. This means that the grinding procedures altered the concentrations of the SRL binding centers available for adsorption, but not the value of the heat of interaction with the SRL molecules, i.e., their chemical nature did not change. In BP, the *K_L_* values (or the heat of adsorption of SRL), on the contrary, increased from C1 to CP2, i.e., with a decrease in the size of AC particles and an increase in the density of oxygen-containing subgroups on the surface. This can be explained by the fact that the functionalization of the AC surface, caused by grinding, probably changes its interaction with BP components; as a result, there was some kind of cooperative effect in the adsorption of these components and SRL on the surface of AC particles, due to which, the *K_L_* value increased (although in general, compared with PBS, it still remained quite low). On the other hand, SRL is known to have high affinity with plasma biomolecules, e.g., human serum albumin (HSA). HSA concentration in blood plasma is 35–50 g/L [28], and its affinity to SRL was determined to be 3.99 × 10^5^ M^−1^ [29]; thus, it can bind SRL and weaken its binding with AC or increase its release from AC. An important conclusion from the consideration of the regularities of the sorption of SRL on various AC fractions in PBS and BP is that the high-molecular components of BP do not so much block the pores of the AC for the access of SRL, but rather reduce the binding constants of the SRL molecules with adsorption centers.

### 3.3. SRL Release Studies

**Figure 7:**
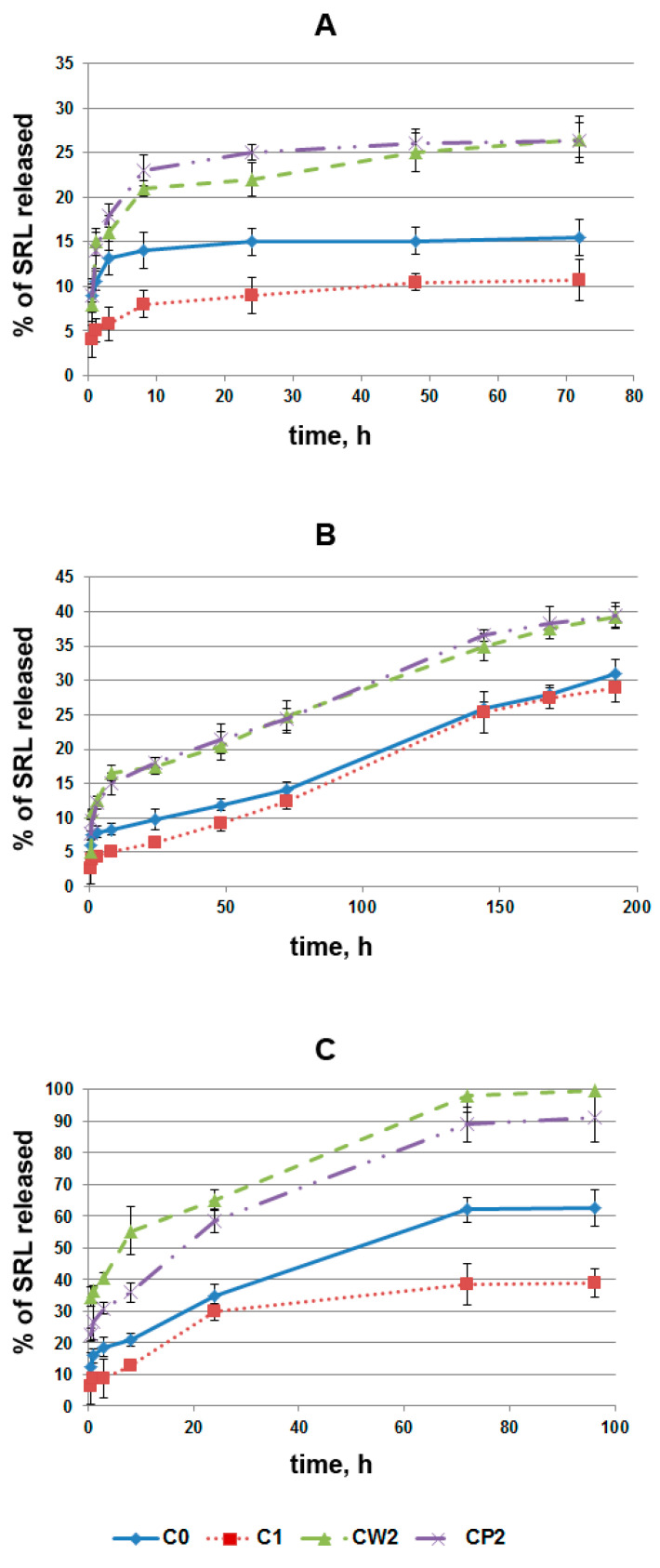
The kinetic curves of SRL release. (**A**) Adsorption and release in PBS; (**B**) adsorption in PBS and release in BP; (**C**) adsorption and release in BP. For adsorption of SRL, the initial mixtures of 1 mL, containing 100 μg of AC and 90 μg of SRL, were incubated for 48 h. Then, the supernatant was removed, and fresh PBS or BP was added. The amount of SRL adsorbed per 100 μg of AC in PBS was 76 ± 5, 83 ± 6, 69 ± 4.6, and 65 ± 4.5 μg for samples C0, C1, CW2, and CP2, respectively. Mean values for adsorption in BP were 6.5 ± 0.7, 15 ± 1.2, 3.6 ± 0.6, and 3.8 ± 0.5 μg per 100 μg of AC for samples C0, C1, CW2, and CP2, respectively.

SRL release studies were performed with PBS and BP. Figure 7 shows kinetic curves of SRL release from various AC fractions, depending on the conditions of SRL pre-adsorption. Small fractions of CW2 and CP2 showed a higher percentage of SRL release in BP, in comparison with large fractions of C0 and C1, obviously due to the low SRL adsorption capacity of CW2 and CP2 carbon in BP (Figure 4). It should be taken into account that the initial loading of SRL was different for all AC fractions. It can be seen that the binding of SRL with different fractions of AC in PBS did not differ drastically, as was the case in BP; the maximal difference was around 20%. In contrast, BP binding of SRL with C0 was almost twice as high as in the CW and CP fractions, while in AC, it exceeds CW and CP nearly fourfold. The C1 fraction was the most effective binder of SRL; clearly, the structures necessary for binding persisted in this fraction, while their accessibility increased during milling.

Release of SRL from AC depends on the binding conditions (Figure 7) and the medium from which the drug is released. When SRL was adsorbed onto AC in PBS, approximately 10–25% of bound SRL was released for 3 days (Figure 7A,B). However, SRL release in PBS was faster than in BP, confirming the importance of the BP–AC interaction in this process, and the fast-forming interaction of BP molecules with AC, whereas the interaction of SRL with surrounding BP biomolecules appears less significant. When SRL is adsorbed in PBS and released in BP, the kinetic curves reach a plateau within 8 days. Maximal SRL release under these conditions was 40% for small fractions of AC (Figure 7B). When SRL is adsorbed onto AC in BP, the kinetic curves reach the plateau within 3 days, and the portion of the released drug reaches 65%, 40%, 95%, and 100% for samples C0, C1, CW2, and CP2, respectively (Figure 7C). Thus, SRL pre-adsorbed in BP was released faster and more completely as compared to those bound with AC in PBS (compare Figure 7B,C). These differences can be explained by two factors: (1) the amount of SRL loaded (depending on samples, SRL adsorption in PBS was 5.5 to 19 times higher than in BP), and (2) the aforesaid capability of plasma biomolecules to prevent SRL binding with high-affinity SRL binding sites, especially on CW2 and CP2, as well as on C0 fractions.

An advantage of using small AC fractions for controlled SRL delivery is reversible drug binding in BP. When loading 3–15 μg of SRL per 100 μg of AC, SRL was released almost completely from CW2 and CP2 for 3 days (Figure 7C), while, for fractions C0 and C1, the kinetic curves reach a plateau at the level of 63% and 40%, respectively. A combination of different AC fractions, and the binding of SRL or similar drugs under different conditions, can obtain multiple variants of drug delivery, as well as drug removal, from biological fluids.

In order to determine the SRL release mechanism from AC, the drug release data were fitted to the four kinetic models most commonly applied in drug release studies: as zero-order, first-order, Higuchi model, Korsmeyer–Peppas model. The highest values of the square of the correlation coefficient (*r*^2^) were detected for the Higuchi and the Korsmeyer–Peppas models (Table 3). Therefore, it can be assumed that SRL release is controlled by diffusion.

## 4. Conclusions

We studied the properties of different fractions of activated carbon after two-stage grinding. The samples obtained were characterized by dynamic light scattering, SEM, and XPS analyses. The SEM data confirm that grinding decreases the size of AC particles. After grinding with a ball mill, a fraction with a particle size of 100–10,000 nm was obtained, and of 100–300 nm after grinding with a bead mill. Small AC particles are prone to aggregation. Polyvinylpyrrolidone was added to prevent the aggregation of carbon particles. It was shown that grinding decreases the size of AC particles and also changes the number of functional groups on the surface, while the number of oxygen-containing functional groups on the surface of AC increases. Fraction C1, obtained after grinding with a ball mill, shows maximal SRL adsorption both in PBS and BP, in comparison with other fractions. Adsorption in PBS was significantly higher than in BP for all fractions. First, components of BP can bind SRL molecules and prevent their adsorption. Second, plasma biomolecules can compete with SRL for adsorption onto AC. The adsorption process depends on SRL concentration. At low SRL concentrations, the adsorption can be described by the Freundlich model. At high concentrations, the data obtained are consistent with the Langmuir model. Fractions C0 and C1 have high adsorption capacity and strongly bind SRL molecules and release less SRL. The rate of SRL release depends on the binding conditions and the composition of the surrounding medium. A combination of different AC fractions, and the binding of SRL or similar drugs under different conditions, can obtain multiple variants of drug delivery, as well as drug removal, from biological fluids. Small AC particles retain the capability to bind SRL, and thus, could be packed into electrospun fibers. The binding/release of SRL by AC in such fibers must be additionally studied, as fiber nanoporosity interferes with the penetration of BP biomolecules and their interaction with AC.

It should be mentioned that other AC particles, such as those produced by the hard-templating method, including hollow carbon nanocapsules and nanocapsules with porous shell [3], could be used for SRL absorption and release, as well as for packaging into electrospun produced fibers. That said, the production of such AC particles and their characterization is a laborious process compared to milling, and the capacity of such particles for SRL needs to be studied.

An AC-enriched matrix is considered as a drug delivery layer, or as a drug accumulating layer, in the electrospinning-produced coating of metal stents (min/max diameter, 2.0/3.5 mm; length, 25 mm). The weight of such ES-produced coatings is no more than 10 mg, and with no more than 10% contributed by AC (thus, 1 mg of AC in total). Nanoparticles 50–200 nm in size were shown to be eliminated through hepatobiliary elimination [30]. The average degradation time for PCL is about 2–3 years [31]. Minor amounts of AC, the absence of AC particle toxicity, combined with slow PCL fiber degradation, allows us to hope that activated carbon will not have any negative effects, even if it is not effectively eliminated from the body. Future in vivo studies should clarify this issue.

## Figures and Tables

**Table 1:** Composition of elements in AC fractions according to XPS.

	O/C	O */C	P/C	*n*/C	Zr/C
C0	0.0500	0.0500	0.0038		
C1	0.0700	0.0700	0.0040	0.0030	
CW2	0.1300	0.1000	0.0030	0.0060	0.0100
CP2	0.1300	0.0800	0.0036	0.0160	0.0150

* With the exception of oxygen in the composition of zirconium oxides and polymers.

**Table 2:** The parameters calculated from the adsorption isotherms of SRL onto AC fractions according to the Freundlich and Langmuir models.

**Freundlich Isotherm (Low SRL Concentrations)**							
**Adsorption in PBS**				**Adsorption in BP**			
**AC Fraction**	**1 */n***	***K_F_* (mg/g (L/mg)^1/*n*^)**	***r* ^2^**	**1 */n***	***K_F_* (mg/g (L/mg)^1/*n*^)**	***r* ^2^**	
C0	1.67	374	0.992	1.33	6.7	0.992	
C1	1.55	440	0.980	1.18	12.2	0.868	
CW2	1.39	192	0.978	1.48	1.0	0.992	
CP2	1.41	129	0.976	1.20	1.2	0.988	
**Langmuir Isotherm (High SRL Concentrations)**							
	**Adsorption in PBS**				**Adsorption in BP**		
**AC Fraction**	***q_max(exp)_* (mg/g)**	***q_max(calc)_* (mg/g)**	***K_L_* (L/g)**	***r* ^2^**	***q_max(calc)_* (mg/g)**	***K_L_* (L/g)**	***r* ^2^**
C0	1850	2000	0.0308	0.965	2000	0.0028	0.989
C1	2840	2500	0.0506	0.984	2500	0.0039	0.996
CW2	1780	1666	0.0275	0.968	1213	0.0070	0.954
CP2	1390	1566	0.0295	0.982	1111	0.0067	0.981

**Table 3:** The values of *r*^2^ for different kinetics models of SRL release from AC.

**Adsorption and Release in PBS**				
	**Zero-Order**	**First-Order**	**Higuchi**	**Korsmeyer–Peppas**
C0	0.528	0.534	0.706	0.883
C1	0.776	0.783	0.918	0.981
CW2	0.674	0.703	0.815	0.851
CP2	0.501	0.585	0.748	0.877
**Adsorption in PBS and RELEASE in BP**				
	**Zero-Order**	**First-Order**	**Higuchi**	**Korsmeyer–Peppas**
C0	0.956	0.960	0.964	0.919
C1	0.979	0.980	0.957	0.926
CW2	0.777	0.806	0.887	0.859
CP2	0.883	0.899	0.975	0.996
**Adsorption and Release in BP**				
	**Zero-Order**	**First-Order**	**Higuchi**	**Korsmeyer–Peppas**
C0	0.953	0.497	0.982	0.928
C1	0.857	0.750	0.957	0.942
CW2	0.931	0.668	0.971	0.944
CP2	0.938	0.539	0.973	0.942

## Supplementary Materials

The following supporting information can be downloaded at: https://www.mdpi.com/article/10.3390/pharmaceutics14071386/s1, Figure S1: The adsorption of SRL onto different fractions of AC. Solutions of SRL with various SRL content amounts in 0.5 mL PBS or BP were incubated with 33 μg of AC for 15 h. The curves present the mass of SRL adsorbed depending of the initial amount of SRL in the solution. The mixtures contained 33 μg of carbon and 20, 60, 90, 150 and 220 μg of 3H-SRL in 0.5 mL PBS or BP. This figure upgrade the data presented in Figure 5 as a percentage of SRL absorbed.
