# Supplementary material for: Activated Carbon for Drug Delivery from Composite Biomaterials: The Effect of Grinding on Sirolimus Binding and Release
Source: Pharmaceutics. 2022 Jun 30;14(7):1386. doi: 10.3390/pharmaceutics14071386 (PMC9325110; doi:10.3390/pharmaceutics14071386)
Supplement: Supplementary file 1 [file pharmaceutics-14-01386-s001.zip › pharmaceutics-1762297-supplementary.pdf]

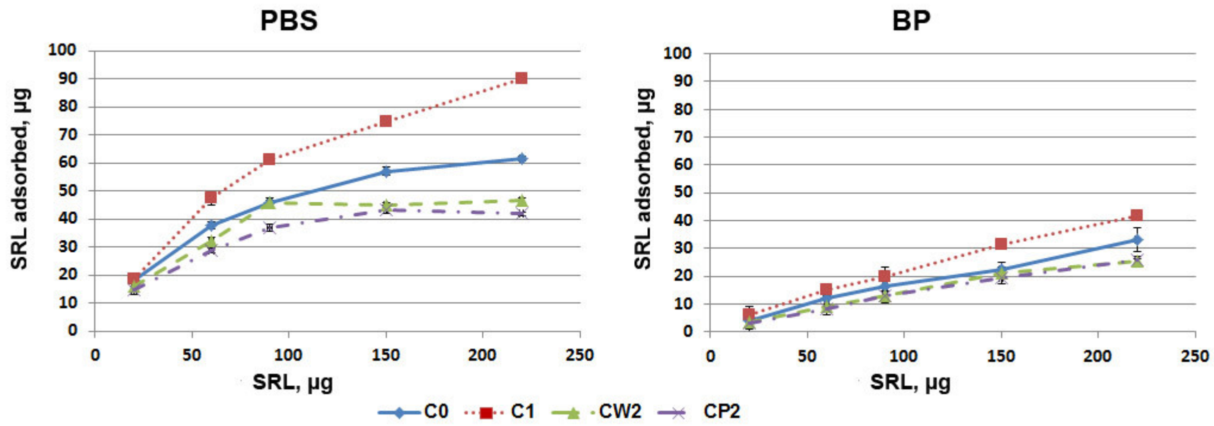

Figure S1. The adsorption of SRL onto different fractions of AC. Solutions of SRL with various SRL content amounts in 0.5 ml PBS or BP were incubated with 33 µg of AC for 15 h. The curves present the mass of SRL adsorbed depending of the initial amount of SRL in the solution. The mixtures contained 33 µg of carbon and 20, 60, 90, 150 and 220 µg of ^{3}H-SRL in 0.5 mL PBS or BP. This figure upgrade the data presented in Figure 5 as a percentage of SRL absorbed.
